# Surface-based morphometry reveals divergent aging trajectories in veterans with and without traumatic brain injury

**DOI:** 10.1007/s11357-025-01893-2

**Published:** 2025-09-29

**Authors:** Michelle C. Eliason, Amrutha Bindu Nagella, Cristian Cuadra, David S. Wack, Ghazala T. Saleem

**Affiliations:** 1 University at Buffalo, Rehabilitation Science, Buffalo, NY USA; 2 Department of Rehabilitation Science, School of Public Health and Health Professions, University at Buffalo, Buffalo, NY USA; 3 Exercise and Rehabilitation Sciences Institute, School of Physical Therapy, Faculty of Rehabilitation Sciences, Universidad Andres Bello, 7591538 Santiago, Chile; 4 Veteran’s Administration Western New York Healthcare System - Veteran’s Hospital, 3495 Bailey Ave, Buffalo, NY USA

**Keywords:** Traumatic brain injury, Cortical thickness, Surface area, Neurodegeneration, Aging

## Abstract

This study investigates how traumatic brain injury (TBI) alters cortical aging by comparing cortical thickness (CT) and surface area (SA) in 34 brain regions between TBI survivors and age-matched controls. Using a cross-sectional retrospective design, 105 Vietnam Veterans (32 with moderate-to-severe TBI, 73 controls) were analyzed via surface-based morphometry. Principal Component Analysis (PCA) reduced dimensionality, and Multivariate Analysis of Covariance tested group differences while controlling for age, education, depression, Post-Traumatic Stress Disorder, and intracranial volume. Findings revealed divergent morphometric signatures of aging: the proportion of SA variance explained by the first principal component (PC1) was lower in the TBI cohort compared to controls, particularly in parietal and limbic regions. Conversely, CT variance explained by PC1 was higher in TBI compared to controls, with fewer factor loadings in frontal and occipital regions, suggesting differential structural aging patterns due to TBI. Regression analysis demonstrated a stronger association of SA with age in TBI (R^2^ = 0.619, p = 0.01), while CT exhibited significant negative age-related thinning in TBI-specific regions (R^2^ = 0.450, p < 0.001). Together, these results suggest that TBI survivors exhibit structured, yet distinct, cortical remodeling, contrasting with the more diffuse patterns seen in normal aging. The differentiated organization of brain areas based on CT and SA points to brain morphology-based biomarkers capable of distinguishing pathological from normative aging trajectories. These biomarkers hold translational potential for refining diagnostic models of brain age and informing targeted neuromodulation or rehabilitation strategies to support cognitive and functional resilience in older adults with TBI.

**Graphical Abstract:**

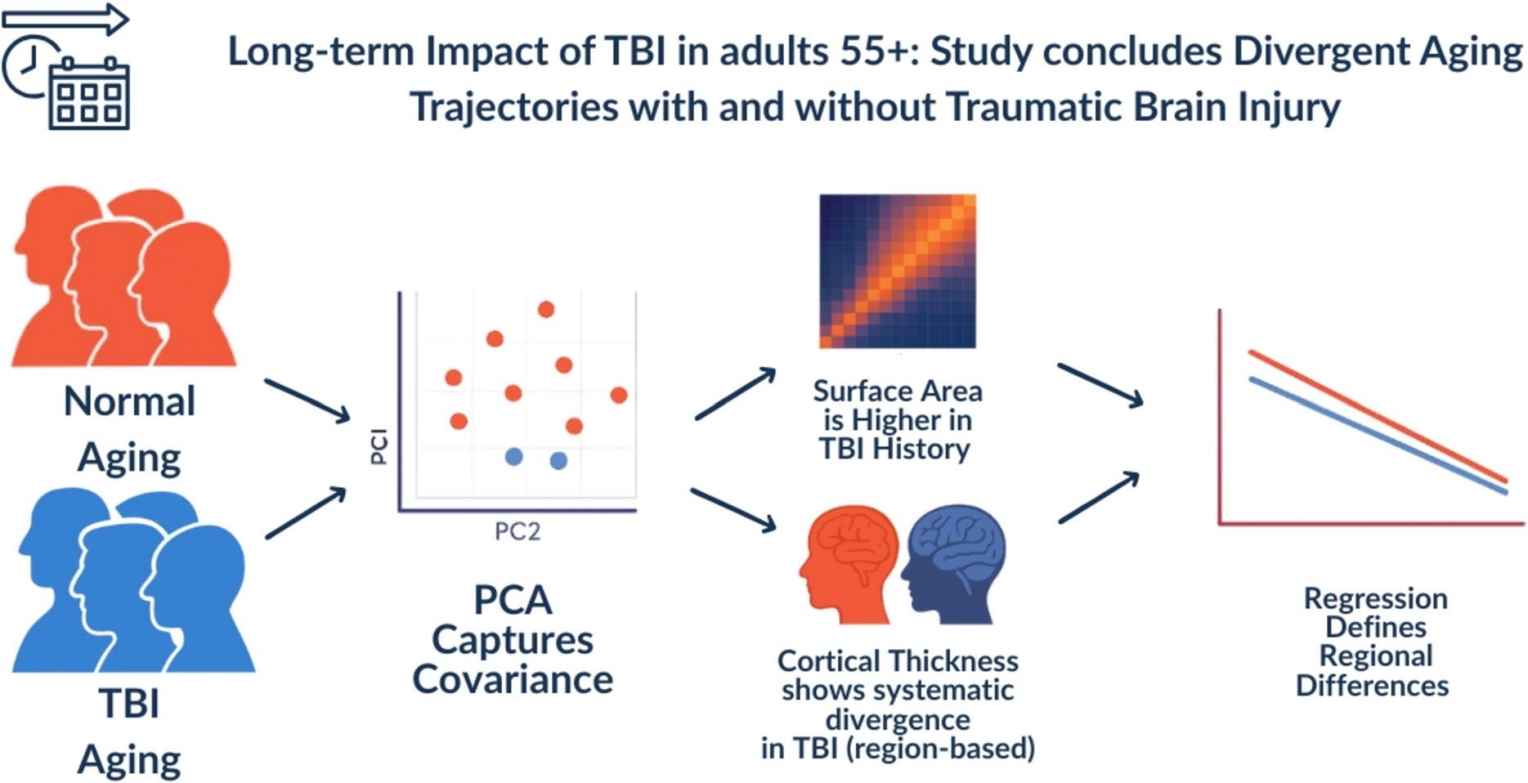

## Introduction

Understanding the long-term effects of moderate to severe Traumatic Brain Injury (TBI) is critical for improving the clinical management of this chronic disease [1, 2] (Full abbreviation list in Table 1). While research has examined the acute effects of TBI on young adults [3, 4], its long-term impact on neurodegeneration and cognitive decline in individuals aged 50 and older remains underexplored [5–7]. The brain's resilience, which refers to its capacity to maintain function despite aging and disease, in relation to TBI must be understood to inform and guide the treatment and prognosis of this disease in aging adults [8]. Structural changes following TBI, including reduced white matter [9] and widespread cortical thinning [9–11], contribute to accelerated brain atrophy and prolonged neuroinflammation [12]. This chronic inflammatory response compromises the blood–brain barrier [13] and impairs key neuronal processes, such as long-term potentiation (i.e. the mechanism that strengthens communication between neurons), dopamine signaling, calcium signaling, and synaptogenesis [2]. This inflammatory response triggers structural adaptation within the brain to maximize efficiency and attenuate deleterious TBI effects, including loss of sensorimotor function, cognitive decline, and reduced quality of life [13, 14].

**Table 1:** Abbreviations and description for key abbreviations throughout the text

Abbreviation	Full Term
TBI	Traumatic Brain Injury
CT	Cortical Thickness
SA	Surface Area
CV	Cortical Volume
ICV	Intracranial Volume
SBM	Surface-Based Morphometry
DMN	Default Mode Network
CRUNCH	Compensation-Related Utilization of Neural Circuits Hypothesis
STAC	Scaffolding Theory of Aging and Cognition
STAC-R	Revised Scaffolding Theory of Aging and Cognition
AD	Alzheimer’s Disease
ADNI	Alzheimer’s Disease Neuroimaging Initiative
NIA	National Institute on Aging
NIBIB	National Institute of Biomedical Imaging and Bioengineering
FDA	Food and Drug Administration
MRI	Magnetic Resonance Imaging
PET	Positron Emission Tomography
MCI	Mild Cognitive Impairment
MMSE	Mini-Mental State Examination
CAPS	Clinician Administered PTSD Scale
PTSD	Post-Traumatic Stress Disorder
MoCA	Montreal Cognitive Assessment
GDS	Geriatric Depression Scale
PCA	Principal Component Analysis
KMO	Kaiser–Meyer–Olkin
MSA	Measure of Sampling Adequacy
PC	Principal Component
MANCOVA	Multivariate Analysis of Covariance
MeFF	Modified Effective Number of Independent Tests

In this study, surface-based morphometry (SBM) is used to examine the atrophy of grey and white matter in both cortical and subcortical structures among male Vietnam War Veterans aged 60 to 85 years, as this method is particularly sensitive to age-related cortical changes such as cortical folding and thickness [15]. Notably, the structural adaptation of grey matter, linked to cortical thickness (CT), and white matter, associated with surface area (SA), significantly contribute to the brain age gap [16–18]. The brain age gap differentiates chronological age (i.e., years since birth) from biological age, which reflects genetics, lifestyle, environment, and pathology. Investigating this distinction allows us to assess whether TBI accelerates neurodegeneration or contributes to additional aging-related risk factors [19]. While Cortical volume (CV) is the product of SA and CT at each location across the cortical mantle [15, 20], combining the independent contributions of SA and CT into a single variable like CV can dilute the distinct effects of each component within a model [21]. This distinction is particularly important in the study of cortical morphology, where SA and CT represent genetically and phenotypically independent properties of the brain [22], and understanding their distinct and independent contributions can help disentangle how early-life factors and lifelong exposures interact to shape brain structure and vulnerability to injury.

SA is associated with the number of ontogenetic columns—structural units of neurons– in each cortical region, while CT reflects the number of neurons stacked within each column; Both SA and CT are primarily determined during neonatal brain development through genetic and early environmental influences [23, 24]. Panizzon et al. (2009) studied the genetic influences of cortical SA and CT in twin men who lived during the Vietnam Era and found similarities at the lobar and regional levels of analysis where total SA and average CT were both highly heritable (0.89 and 0.81, respectively) but were essentially unrelated genetically (r = 0.08). Of note, total intracranial volume (ICV) is also highly heritable, with genetic factors accounting for 78% of its variance, however while ICV strongly correlates with total SA (r = 0.81, p < 0.0001), it does so weakly with CT (r = 0.15, p < 0.001) [20]. These distinct structural features—SA and CT—define brain morphology and have been shown to exhibit differential trajectories across the lifespan, offering insight into how age-related changes in brain morphology may influence broader patterns of network function. A study examining CT correlations across 78 regions found that, although brain networks overlapped between young and older adults, aging led to a decline in network efficiency, characterized by weaker separation between functional regions (i.e., lower modularity). The lower modularity, has resulted in increased unintended connectivity between areas that should operate independently [10]. This effect was most pronounced in networks responsible for higher-order cognition (strategic/executive module, p = 0.005) and intrinsic functional organization (default mode network (DMN) module, p = 0.035). Disruptions in the DMN and sensorimotor/spatial modules were notable, each showing increased inter-modular connectivity in five regions. Additionally, twelve regions across all modules exhibited significantly altered and evenly distributed intra-modular connectivity, suggesting a broader, coordinated decline in cognitive function associated with normal aging [10]. These findings align with theoretical models of aging, such as Compensation-Related Utilization of Neural Circuits Hypothesis (CRUNCH) and Scaffolding Theory of Aging and Cognition (STAC), which propose that increased inter-modular connectivity may reflect both compensatory neural recruitment and a decline in network efficiency as cognitive demands increase [14]. The CRUNCH model suggests that as cognitive tasks become more demanding, older adults and individuals with neurological injury (e.g., TBI) over-recruit additional brain regions to maintain performance [25]. This over-recruitment may initially support cognition but eventually reaches a limit, leading to cognitive decline. Similarly, STAC and STAC-R propose that the brain builds compensatory scaffolding by activating alternative circuits to offset structural deterioration [26, 27]. However, this scaffolding is not always optimal—while some individuals maintain preserved brain function through brain maintenance mechanisms such as adaptive shifts in activation patterns and compensatory recruitment of alternative neural circuits to sustain performance [26]**,** others experience increased reliance on inefficient compensatory pathways due to accumulated neural insults, stress, or other life-course factors [26, 27].

Because TBI is a leading environmental risk factor for neurodegeneration, including Alzheimer’s Disease (AD) and other forms of cognitive decline [13], distinguishing its structural impact from normal aging is essential. Analyzing SA and CT separately provides a more precise framework for understanding the variability in aging trajectories post-TBI, as SA’s strong genetic heritability and its correlation with ICV contrast with CT’s greater susceptibility to environmental and pathological influences [28–30]. These morphometric distinctions allow for a more refined investigation of how TBI accelerates or alters cortical aging beyond expected neurodegenerative processes (i.e., senescence). By leveraging SBM to examine these structural patterns regionally, this study aims to advance research on the structural markers that differentiate biological aging in TBI survivors from their age-matched counterparts, informing future neuroimaging methodologies and research on targeted interventions for the aging population [16].

The primary aim of this study is to examine the impact of TBI on morphometric brain characteristics compared to healthy aging across 34 brain regions in each hemisphere. The first hypothesis is that TBI survivors (n = 32) will exhibit distinct CT and SA variability patterns compared to age-matched healthy controls (n = 73), as revealed through principal component analysis. A secondary aim focuses on investigating the relationship between SA and CT as it relates to the brain-age gap. The hypothesis posits that age will significantly explain the variability among SA and CT of TBI survivors (n = 32) compared to age-matched healthy controls (n = 73), indicating a distinction between biological and chronological age.

## Methods

### Alzheimer’s disease neuroimaging initiative (ADNI)

Data used to prepare this article were obtained from the Alzheimer's Disease Neuroimaging Initiative (ADNI) database (adni.loni.usc.edu). The ADNI was launched in 2003 by the National Institute on Aging (NIA), the National Institute of Biomedical Imaging and Bioengineering (NIBIB), the Food and Drug Administration (FDA), private pharmaceutical companies and non-profit organizations, as a $60 million, 5-year public–private partnership. The primary goal of ADNI has been to test whether serial magnetic resonance imaging (MRI), positron emission tomography (PET), other biological markers, and clinical and neuropsychological assessment can be combined to measure the progression of mild cognitive impairment (MCI) and early Alzheimer's disease (AD). The Principal Investigator of this initiative is Michael W. Weiner, MD, VA Medical Center and University of California—San Francisco. ADNI is the result of efforts of many co-investigators from a broad range of academic institutions and private corporations, and subjects have been recruited from over 50 sites across the U.S. and Canada. For up-to-date information, see www.adni-info.org.

### Inclusion and exclusion criteria

Eligible participants for the study included Vietnam War veterans aged 50 to 85 residing within 150 miles of an ADNI clinic. The dataset for this study included a total of 105 Vietnam Veterans, with 32 (30.48%) having a history of moderate to severe TBI during military service without clinical diagnosis of PTSD and 73 (69.52%) age-matched, healthy controls (Table 2). Those recruited into the TBI cohort had a documented history of moderate to severe non-penetrating TBI during military service, with loss of consciousness or altered mental status exceeding 24 h. The participants in the control cohort had no history of significant head trauma, with any loss of consciousness under five minutes. All participants had intact cognitive function (MMSE 24–30) and no signs of clinical dementia, psychosis, or severe mental health conditions. Exclusion criteria for TBI and Control cohorts included Post Traumatic Stress Disorder (PTSD) diagnosis (CAPS > 30), and significant neurological conditions, such as stroke or seizure disorders. Participants with unstable medical conditions, contraindications to Magnetic Resonance Imaging (MRI) or lumbar puncture, and recent investigational drug use were excluded from the study [31].

**Table 2:** Descriptive summary of continuous variables comparing TBI and Control cohorts. The 3rd column represents 2-sample t-test (Education, Age, GDS, CAPS, ICV) and Mann Whitney U (MoCA) outcomes

Demographic Descriptive Summary					
		**Control**	**TBI**	**Mann–Whitney or T-test**	**p-value**
Education	Education mean	16.09	16.03	W = 1093.5	0.94
	Education sd	2.13	2.46		
	Education median	16.00	16.00		
	Education IQR	4.00	4.00		
Age	Age mean	71.16	69.47	W = 906	0.15
	Age sd	5.91	5.32		
	Age median	70.00	68.00		
	Age IQR	8.00	6.25		
GDS	GDS mean	0.78	1.25	W = 1172.5	0.59
	GDS sd	0.95	1.76		
	GDS median	0.00	0.00		
	GDS IQR	1.00	2.00		
CAPS	CAPS mean	1.78	5.44	W = 1494	0.001
	CAPS sd	3.58	6.26		
	CAPS median	0.00	3.00		
	CAPS IQR	2.00	10.25		
ICV	ICV mean	1526591	1553511	t = 0.95	0.35
	ICV sd	135964	128676		
	ICV median	1538710	1564435		
	ICV IQR	196660	203700		
**Categorical Variables Descriptive Summary**					
**Cohort**	**Count**	**Percentage**			
TBI	32	30.48%			
Control	73	69.52%			
Total	105				
**Handedness**					
Right-handed	97	92.38%			
Left-handed	8	7.62%			
Total	105				

### Behavioral assessments

All participants enrolled in the study underwent behavioral assessment. The Clinician Administered PTSD Scale (CAPS) is a 30-item structured interview designed to assess PTSD symptoms and their severity during the last month [32]. It has strong reliability (Cronbach’s alpha > 0.90) and convergent validity with other PTSD measures [33]. Each item is rated on a 5-point scale (0 = "absent" to 4 = "extreme/incapacitating"), with higher total scores indicating greater symptom severity [32, 33]. The Geriatric Depression Scale (GDS) is a 15-item questionnaire used to screen for depression in older adults. It focuses on identifying depressive symptoms without relying on physical complaints to provide insight into the participant’s mental health status at baseline. [34]. It is highly reliable (Cronbach’s alpha > 0.80) and validated for use in older populations. Responses are scored dichotomously (0 = "no" and 1 = "yes"), with total scores ranging from 0 to 15. A score of 5 or greater is generally used as a cutoff for identifying possible depression, with higher scores indicating greater depressive symptomatology [35]. The following data tables were utilized in this analysis: Central SFVAMC Eligibility, Montreal Cognitive Assessment (MoCA), Demographics, Mini-Mental State Examination (MMSE), Geriatric Depression Scale (GDS), and Clinician Administered PTSD Scale (CAPS).

### Neuroimaging data

Data were obtained using 3 Tesla MRI scanners following standardized procedures across 24 ADNI sites. Specifically, the imaging data in this study is obtained from dataset UCSF—Cross-Sectional FreeSurfer (FreeSurfer Version 5.1) which includes post-processed MRI-values for surface area (SA, mm2) and cortical thickness (CT, mm) Further information on MRI acquisition is available in the DOD ADNI procedures manual [31, 36].

### Statistical methods and data reduction

Statistical analyses were conducted in R (Version 2024.04.2). Figures were generated in R using the ggplot2, factoextra, and psych packages. The statistical approach involved data reduction, preprocessing, and multivariate analyses to examine CT and SA differences between TBI and Control cohorts. Regions spanned frontal, parietal, temporal, occipital, and limbic cortices, including the Paracentral (LPaC, RParaC), Pars Opercularis (LPOp, RPOp), Pars Orbitalis (LPO, RPO), Pars Triangularis (RPT), Postcentral (LPoC, RPoC), Posterior Cingulate (LPC, RPC), Precentral (LPrC, RPrC), Precuneus (LPc, RPc), Rostral Anterior Cingulate (LRAC), Rostral Middle Frontal (LRmF, RRMF), Superior Frontal (LSF, RSF), Superior Parietal (LSP, RSP), Superior Temporal (LST, RST), Supramarginal (Lsupm, Rsupm), Temporal Pole (RTP), Transverse Temporal (RTT), Insula (RI), Banks of the Superior Temporal Sulcus (RBstS), Caudal Anterior Cingulate, Caudal Middle Frontal (LCaMF, RCaMF), Cuneus (RCu), Fusiform (LFu, RFu), Inferior Parietal (LIP, RIP), Inferior Temporal (LIT, RIT), Isthmus Cingulate (LIsC), Lateral Occipital (LLO, RLO), Lateral Orbitofrontal (LLOrbF, RLOrbF), Lingual (LLg), and Medial Orbitofrontal (LMOrbF) (Table 3).

**Table 3:** Abbreviations and description for key Cortical Thickness and Surface Area Variables

Abbreviation	Region Name	Abbreviation	Region Name
LPaC	Left Paracentral	RIT	Right Inferior Temporal
RPOp	Right Pars Opercularis	RSP	Right Superior Parietal
LIT	Left Inferior Temporal	LPO	Left Pars Orbitalis
LFu	Left Fusiform	LSP	Left Superior Parietal
LPrC	Left Precentral	RBstS	Right Bankssts
LCaMF	Left Caudal Middle Frontal	Rsum	Right Supramarginal
RLOrbF	Right Lateral Orbitofrontal	RTP	Right Temporal Pole
LRmF	Left Rostral Middle Frontal	RCu	Right Cuneus
LLO	Left Lateral Occipital	RCaMF	Right Caudal Middle Frontal
RPoC	Right Postcentral	RTT	Right Transverse Temporal
RLO	Right Lateral Occipital	RPT	Right Pars Triangularis
RPc	Right Precuneus	LLOrbF	Left Lateral Orbitofrontal
LPOp	Left Pars Opercularis	LIsC	Left Isthmus Cingulate
LPc	Left Precuneus	RPC	Right Posterior Cingulate
RMdT	Right Middle Temporal	RParaC	Right Paracentral
RSF	Right Superior Frontal	LMOrbF	Left Medial Orbitofrontal
LSF	Left Superior Frontal	RPO	Right Pars Orbitalis
LIP	Left Inferior Parietal	RI	Right Insula
RParaC	Right Paracentral	Rsupm	Right Supramarginal
LST	Left Superior Temporal	LPC	Left Posterior Cingulate
RIP	Right Inferior Parietal	RRMF	Right Rostral Middle Frontal
RFu	Right Fusiform	LRAC	Left Rostral Anterior Cingulate
RST	Right Superior Temporal	RPrC	Right Precentral
LPoC	Left Postcentral	LWM	Left Hemisphere White Matter
Lsupm	Left Supramarginal	RWM	Right Hemisphere White Matter
LLg	Left Lingual		
LMdT	Left Middle Temporal		

Dimensionality reduction was conducted via Principal Component Analysis (PCA), with preprocessing steps ensuring statistical robustness. Kaiser–Meyer–Olkin (KMO) and Bartlett’s test, confirmed sampling adequacy and justified PCA [37–40]. Variables with individual measures of sampling adequacy (MSA) below 0.60 were excluded [41, 42]. To avoid overfitting due to high-dimensional data, preprocessing followed best practices in PCA analysis, standardizing variables to unit variance [29, 43]. Only principal components (PCs) explaining ≥ 10% variance was retained to maintain interpretability (Table 3) [29, 43]. Factors loading onto PC1 were filtered (≥ 0.63) to ensure meaningful contributions [44]. To compare regional contribution between cohorts, scatter plots and regression analysis of PC1 factors were used and Levene’s test confirmed homogeneity of variance to ensure observed differences were not confounded by unequal variance across regions. Outliers exceeding ± 2 standard deviations were excluded to refine the dataset [29, 43].

Multivariate analysis of covariance (MANCOVA) using Pillai’s Trace examined CT and SA differences between cohorts, with cohort (TBI vs. Control) as the independent variable and age, education, GDS, CAPS, and ICV as covariates [45]. Modified Effective Number of Independent Tests (MeFF) was used to control for multiple comparisons which leverages eigenvalue decomposition of the correlation matrix [46] and has been used in neuroimaging [47] to balance statistical power while mitigating false positives [46–48]. This method avoids the overly conservative Bonferroni correction, which may increase Type II error risk [49]. An exploratory regression analysis was conducted on significant variables post-MANCOVA to assess relationships between cortical thickness, surface area, and ICV in TBI and control groups. Scatter plots and linear models quantified regional structural differences.

## Results

### Participants

The final dataset consisted of 105 Vietnam Veterans: 32 (30.48%) with a history of moderate-to-severe TBI during military service and 73 (69.52%) age-matched healthy controls. The total sample was comprised of 97 right-handed (92.38%) participants, while only 7.62% were left-handed. The mean education levels were 16.03 ± 2.46 years for the TBI cohort and 16.09 ± 2.13 years for the control cohort. The average age at exam was 69.47 ± 5.32 years in the TBI cohort and 71.16 ± 5.91 years in the control cohort. GDS scores were 1.25 ± 1.76 for the TBI cohort and 0.78 ± 0.95 for the control cohort, indicating more reported depressive symptoms in the TBI cohort. None of the TBI participants had a clinical diagnosis of PTSD. However, CAPS scores were 5.44 ± 6.26 in the TBI cohort and 1.78 ± 3.58 in the control cohort, reflecting greater PTSD symptomatology among TBI participants. ICV values were 1,553,511 ± 128,676 for the TBI cohort and 1,526,591 ± 135,964 for the control cohort, indicating comparable intracranial volumes between cohorts (Table 2).

To assess cohort differences, normality was inspected using qq-plots and Shapiro–Wilk tests. Independent t-tests were used for normally distributed variables, while Wilcoxon rank-sum tests addressed non-parametric data. No significant differences were found in education (W = 1093.5, p = 0.94), age (W = 906, p = 0.15), GDS scores (W = 1172.5, p = 0.59), or ICV (t = 0.95, p = 0.35). CAPS scores were significantly higher in the TBI cohort (W = 1494, p = 0.001) (Table 2).

### KMO and Bartlett’s test of sphericity

KMO values, 0.67 for SA and 0.86 for CT, were interpreted according to the index of factorial simplicity and classified as “middling” and “meritorious” respectively [40]. Bartlett’s tests were highly significant for both SA (χ^2^ = 15,156.06, df = 2415, p < 0.001) and CT (χ^2^ = 12,411.84, df = 2278, p < 0.001), confirming the presence of correlations between variables necessary for factor analysis.

### Principal component analysis and regression analysis

#### Surface area

**Fig. 1:**
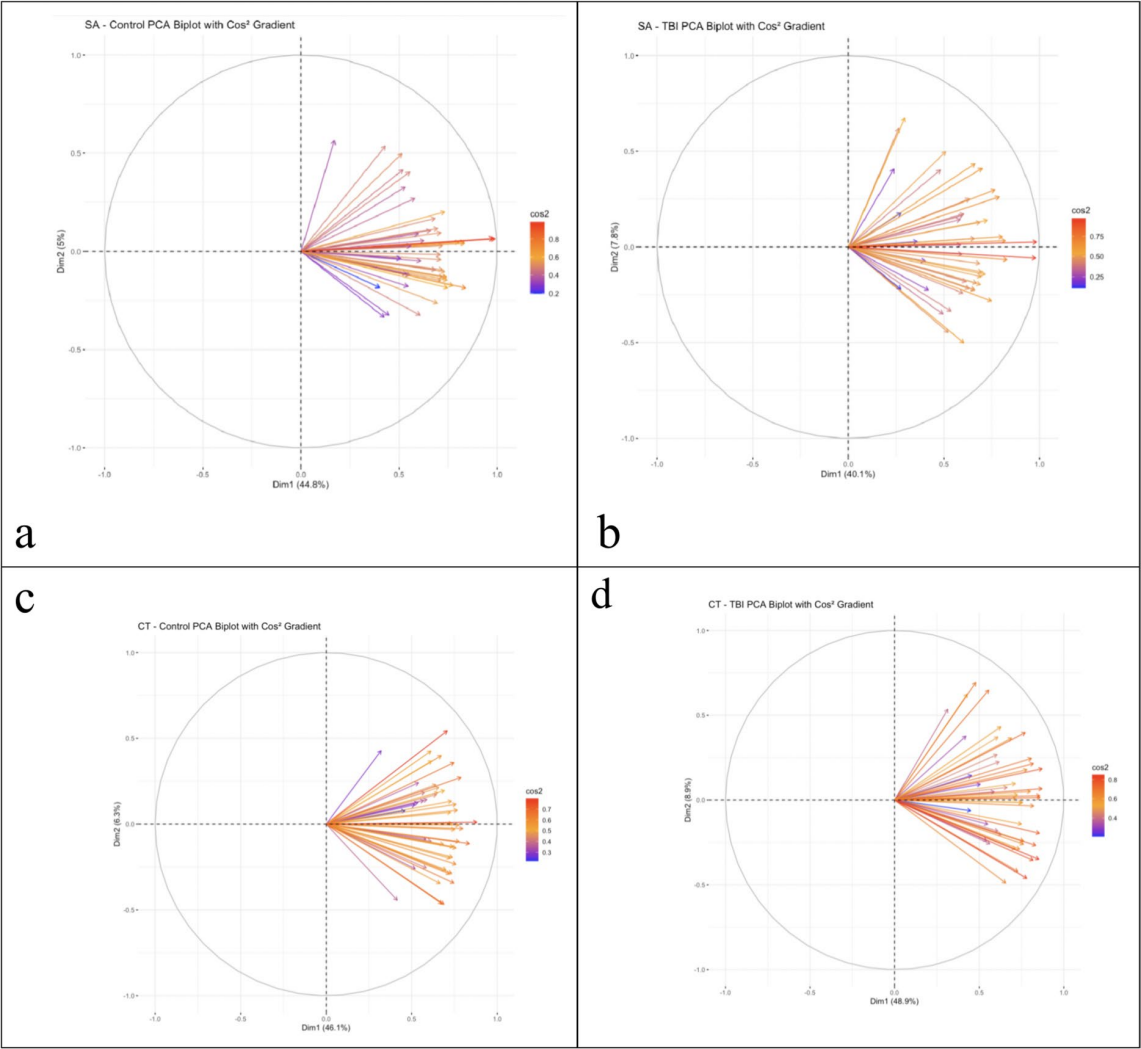
Panels A and B display the unfiltered principal component (PC1 and PC2) biplot vectors for Surface Area (SA) from Principal Component Analysis (PCA). Panels c and d show the unfiltered PC1 and PC2 biplot vectors for Cortical Thickness (CT). Panels a and c represent control data, while Panels b and d represent TBI data. The color gradient indicates Cos^2^ values, reflecting the contribution of each variable to the principal components

**Fig. 2:**
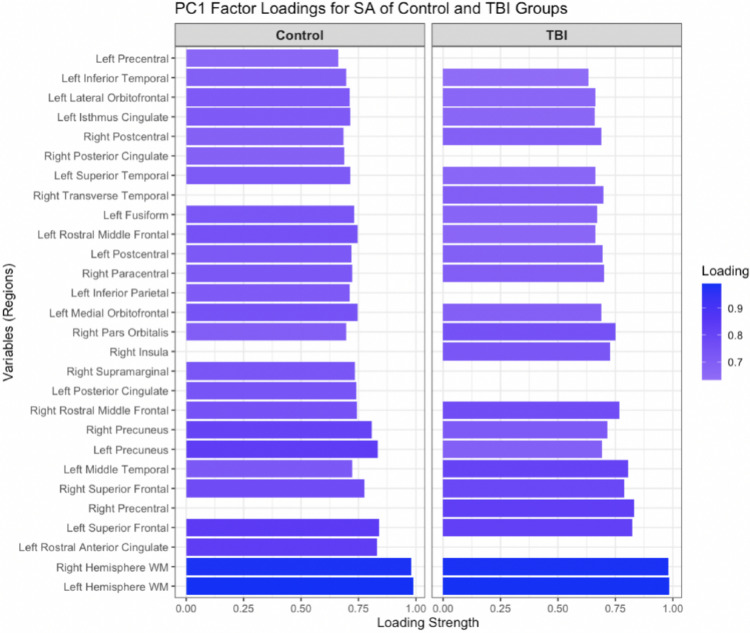
PC1 Factor Loadings for Surface Area in Control and TBI Groups. This bar plot compares the factor loadings of cortical surface area for the first principal component (PC1) between Control and TBI cohorts. The x-axis represents loading strength, while the y-axis lists brain regions contributing to PC1. Bars are color-coded by loading magnitude, with darker shades indicating higher loadings

In SA, the first principal component (PC1) accounted for 44.8% of the variance in the Control cohort and 40.1% in the TBI cohort (Fig. 1), with all other components explaining less than 10% of the variance each. The PCA for the Control cohort yielded 23 factor loadings onto PC1 (Fig. 2), distributed across four cortical regions. Specifically, eight were in the frontal cortex (LRmF, LPrC, RRMF, LSF, RSF, LLOrbF, LMOrbF, RPO), seven in the parietal cortex (RParaC, RPoC, LIP, RPc, Rsupm, LPoC, LPc), four in the temporal cortex (LST, LIT, LFu, LMdT), and four in limbic structures (LIsC, RPC, LPC, LRAC). In contrast, the TBI cohort exhibited only 20 PC1 factor loadings, following a similar distribution pattern. Eight were in the frontal cortex (LRmF, RRMF, LSF, RSF, LLOrbF, LMOrbF, RPO, RPrC), five in the parietal cortex (RParaC, RPoC, LPoC, RPc, LPc), five in the temporal cortex (LST, LIT, LFu, LMdT, RTT), and two in limbic structures (LIsC, RI). While hemispheric white matter (LWM, RWM) demonstrated high component loadings in both groups, it was excluded from regional counts due to its global rather than regionally specific representation.

#### Cortical thickness

**Fig. 3:**
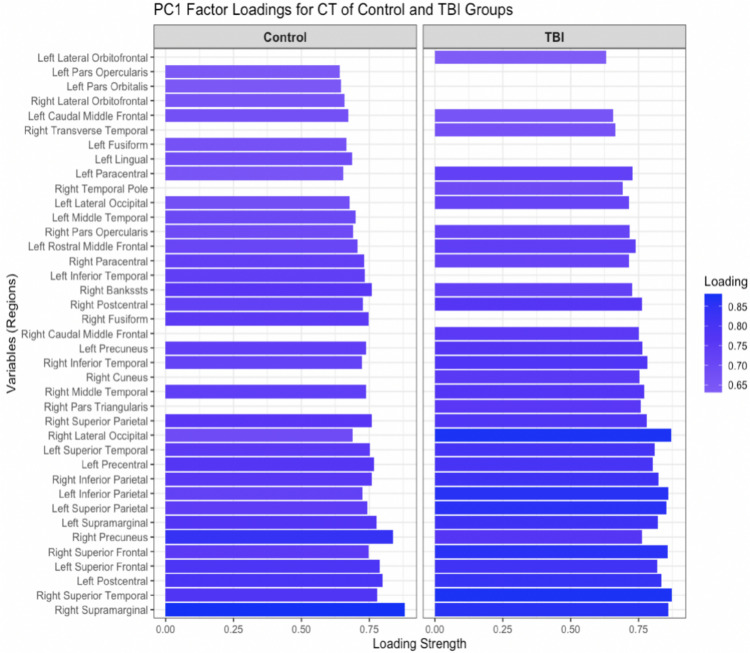
PC1 Factor Loadings for Cortical Thickness in Control and TBI Groups. This bar plot compares the factor loadings of cortical thickness for the first principal component (PC1) between Control and TBI cohorts. The x-axis represents loading strength, while the y-axis lists brain regions contributing to PC1. Bars are color-coded by loading magnitude, with darker shades indicating higher loadings

In CT, the first principal component (PC1) accounted for 46.1% of variance in the Control cohort and 48.9% of variance in the TBI cohort (Fig. 1), with all other components explaining less than 10% of variance each. The Control cohort showed 33 high factor loadings onto PC1(Fig. 3), distributed across four cortical regions. Specifically, nine were located in the frontal cortex (LPO, LSF, RSF, LPOp, RPOp, LRmF, RLOrbF, LCaMF, LPrC), twelve in the parietal cortex (Rsupm, Lsupm, LSP, RSP, LPoC, RIP, RParaC, LPaC, LIP, LPc, RPc, RPoC), eight in the temporal cortex (RIT, LMdT, RST, RFu, LST, RMdT, LFu, LIT), and four in the occipital region (RBstS, LLg, RLO, LLO). In contrast, the TBI cohort exhibited 31 factors loading onto PC1, following a similar distribution pattern. Eight were in the frontal cortex (RPOp, LPrC, LCaMF, RSF, LSF, RCaMF, LLOrbF, RPT), twelve in the parietal cortex (LPaC, RPoC, LPoC, RPc, LPc, LIP, RParaC, RIP, Lsupm, Rsupm, RSP, LSP), seven in the temporal cortex (RMdT, LST, RST, RIT, LIT, RTP, RTT), and four in the occipital region (LLO, RLO, RBstS, RCu).

#### Regression analysis of PC1 factor loadings

**Fig. 4:**
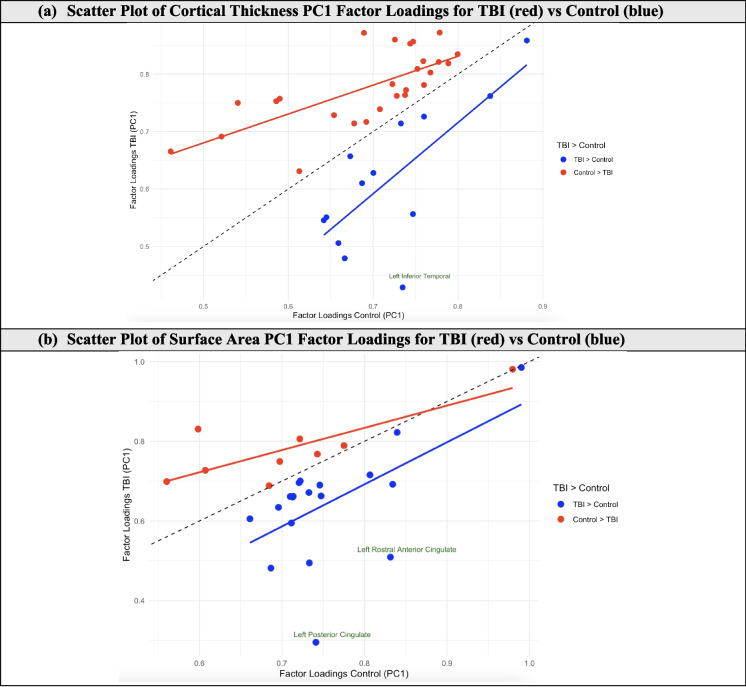
These plots compare Cortical Thickness and Surface Area contributions (PC1 Factor Loading values) between TBI (red) and Control (blue) cohorts where (a) represents Cortical Thickness and (b) represents Surface Area. Points near the diagonal show similar contributions, while red or blue dominance reflects group-specific differences. Regression lines highlight distinct trends, with notable outliers labeled

The scatter plot of PC1 FL values for CT and SA compared the regional variability between TBI (red points) and Control (blue points) cohorts (Fig. 4). Red points represent regions where the FL is higher in the TBI cohort, while blue points indicate regions with higher FL in the Control cohort. For SA, regression analysis demonstrated a weaker association in the Control-Dominant Model (β = 1.056, p = 0.01, R^2^ = 0.328, adj. R^2^ = 0.288, F(1,17) = 8.283), while the TBI-Dominant Model exhibited a stronger association (β = 0.558, p = 0.01, R^2^ = 0.619, adj. R^2^ = 0.564, F(1,7) = 11.36). The residual standard error was lower in the TBI model (0.058) compared to controls (0.119), indicating less dispersion of SA values around the regression line in the TBI group. CT FL values revealed distinct patterns of association. The Control-Dominant Model showed a significant relationship between CT and Control values **(**β = 1.239, p = 0.004, R^2^ = 0.544, adj. R^2^ = 0.502, F(1,11) = 13.1**).** The TBI-Dominant Model exhibited a weaker relationship **(**β = 0.503, p < 0.001, R^2^ = 0.450, adj. R^2^ = 0.478, F(1,24) = 23.93**).** Notably, the residual standard error was lower in the TBI model **(**0.046**)** compared to the control model **(**0.087**).** Outlier analysis, conducted using z-scores, identified one outlier in CT and two in SA. Specifically, the LIT region exhibited a z-score of 3.04 in CT, while the LPC and LRAC exhibited z-scores of 3.31 and 2.13, respectively, in SA. Outliers exceeding z-scores of ± 2 were removed prior to the multivariate analysis of covariance (MANCOVA) analysis [29, 43].

## Multivariate analysis of covariance

The MANCOVA results indicated that Age (SA F(1,98) = 2.16, p = 0.004; CT F = 5.80, p = 0.001) and ICV (SA F = 2.27, p < 0.001 and CT F(1,98) = 1.86, p = 0.02) account for a statistically significant proportion of the variance in cortical structures between the TBI and Control cohorts. A post-hoc analysis (Table 4) further identified variables significantly explained by Age and ICV after correcting for multiple comparisons using MeFF**.** The calculated MeFF for SA was 12.22, resulting in an adjusted significance threshold of p < 0.0041, while the MeFF for CT was 16.36, yielding an adjusted significance threshold of p < 0.0031.

**Table 4:** Multivariate Analysis of Covariance Post-Hoc Analysis with Multiplicity Correction Method (MeFF)

MANCOVA Post-Hoc Analysis with Multiplicity Correction										
AGE										
Response	**Hemisphere**	**Region**	**Variable**	**Df**		**Sum Sq**	**Mean Sq**	**F value**	**Original P-value**	**Corrected**
ST38TA	Left	Lingual	Age	1		0.33584	0.33584	22.4802	7.207E-06	Significant
ST40TA	Left	Middle Temporal	Age	1		0.57191	0.57191	25.94	1.708E-06	Significant
ST51TA	Left	Precentral	Age	1		0.32113	0.32113	11.4641	0.001022	Significant
ST85TA	Right	Fusiform	Age	1		0.32167	0.32167	10.1808	0.001907	Significant
ST99TA	Right	Middle Temporal	Age	1		0.43497	0.43497	16.336	0.0001056	Significant
ST51SA	Left	Precentral	Age	1		1,551,872	1,551,872	9.2177	0.003071	Significant
Intracranial Volume (ICV)										
Response	**Hemisphere**	**Region**	**Variable**	**Df**	**Sum Sq**		**Mean Sq**	**F value**	**Original** **P-value**	**Corrected**
ST102SA	Right	Paracentral	ICV	1	1,513,265		1,513,265	51.9936	1.175E-10	Significant
ST105SA	Right	Pars Orbitalis	ICV	1	278,137		278,137	40.665	5.962E-09	Significant
ST108SA	Right	Postcentral	ICV	1	4,261,827		4,261,827	40.7021	5.882E-09	Significant
ST109SA	Right	Posterior Cingulate	ICV	1	667,743		667,743	28.7692	5.444E-07	Significant
ST110SA	Right	Precentral	ICV	1	9,131,936		9,131,936	52.7161	9.244E-11	Significant
ST111SA	Right	Precuneus	ICV	1	7,457,030		7,457,030	52.997	8.424E-11	Significant
ST114SA	Right	Rostral Middle Frontal	ICV	1	25,851,503		25,851,503	87.9456	2.712E-15	Significant
ST115SA	Right	Superior Frontal	ICV	1	31,143,898		31,143,898	107.2299	2E-16	Significant
ST118SA	Right	Supramarginal	ICV	1	6,315,980		6,315,980	34.7578	5.307E-08	Significant
ST121SA	Right	Transverse Temporal	ICV	1	90,482		90,482	31.8707	1.606E-07	Significant
ST130SA	Right	Insula	ICV	1	2,890,372		2,890,372	63.8164	2.684E-12	Significant
ST26SA	Left	Fusiform	ICV	1	4,867,417		4,867,417	42.0523	3.62E-09	Significant
ST28SA	Left	Hemisphere WM	ICV	1	2,805,896,977		2,805,896,977	160.2023	2E-16	Significant
ST31SA	Left	Inferior Parietal	ICV	1	8,962,310		8,962,310	37.7949	1.703E-08	Significant
ST32SA	Left	Inferior Temporal	ICV	1	5,210,202		5,210,202	35.3006	4.322E-08	Significant
ST34SA	Left	Isthmus Cingulate	ICV	1	1,094,684		1,094,684	49.3643	2.84E-10	Significant
ST36SA	Left	Lateral Orbitofrontal	ICV	1	2,721,638		2,721,638	57.1539	2.176E-11	Significant
ST39SA	Left	Medial Orbitofrontal	ICV	1	2,493,999		2,493,999	69.7137	4.531E-13	Significant
ST40SA	Left	Middle Temporal	ICV	1	5,383,752		5,383,752	54.5951	4.983E-11	Significant
ST49SA	Left	Postcentral	ICV	1	6,744,614		6,744,614	32.7092	1.161E-07	Significant
ST50SA	Left	Posterior Cingulate	ICV	1	605,296		605,296	32.1154	1.461E-07	Significant
ST51SA	Left	Precentral	ICV	1	7,288,125		7,288,125	43.2893	2.331E-09	Significant
ST52SA	Left	Precuneus	ICV	1	6,414,133		6,414,133	60.5772	7.34E-12	Significant
ST54SA	Left	Rostral Anterior Cingulate	ICV	1	938,447		938,447	52.4252	1.018E-10	Significant
ST55SA	Left	Rostral Middle Frontal	ICV	1	23,012,529		23,012,529	93.6928	6.007E-16	Significant
ST56SA	Left	Superior Frontal	ICV	1	31,885,856		31,885,856	82.0981	1.321E-14	Significant
ST58SA	Left	Superior Temporal	ICV	1	4,085,857		4,085,857	34.1741	6.625E-08	Significant
ST87SA	Right	Hemisphere WM	ICV	1	3,157,368,898		3,157,368,898	166.7639	2E-16	Significant

**Fig. 5:**
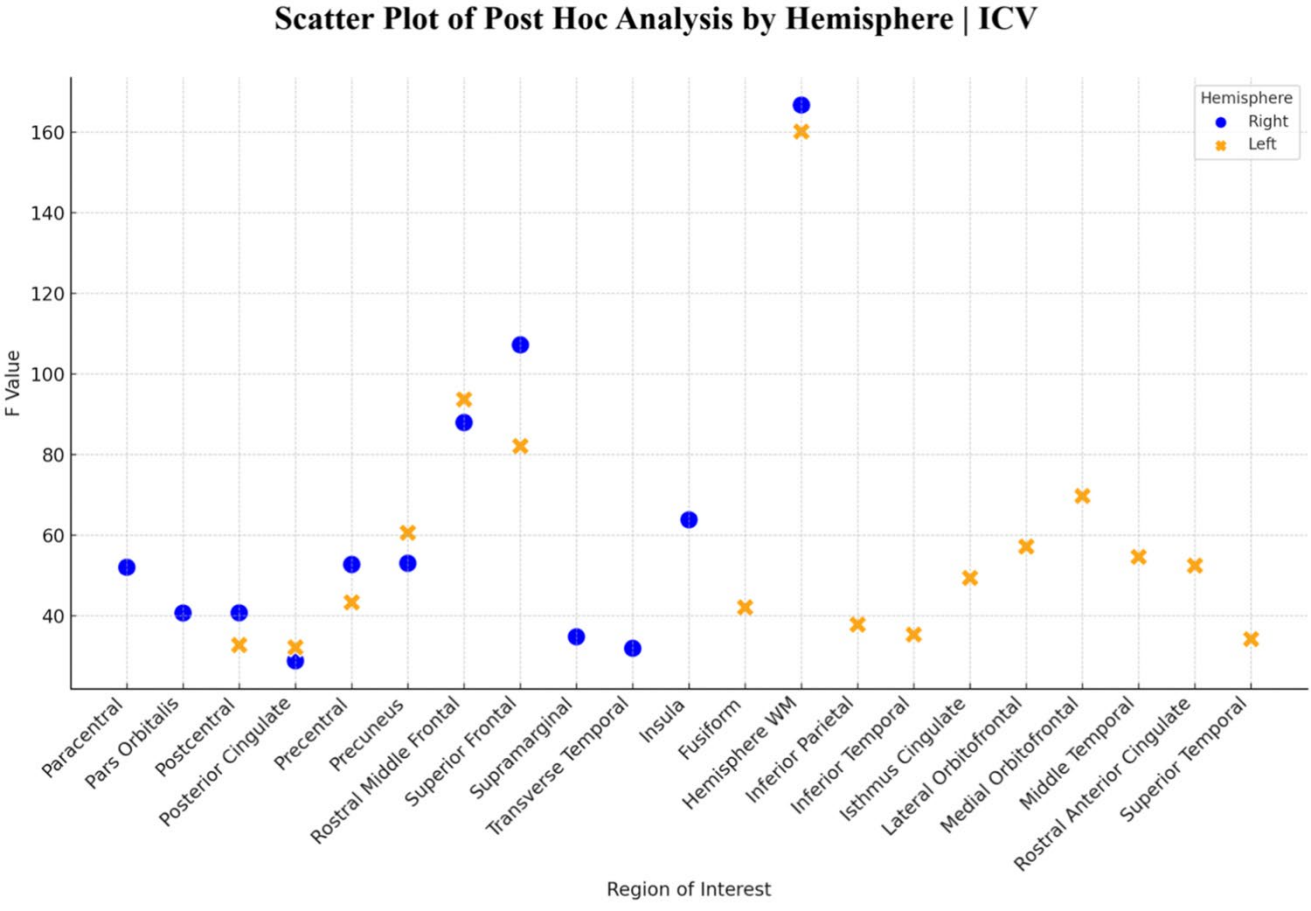
Scatter plot of post hoc analysis results for intracranial volume (ICV) following MANCOVA, stratified by hemisphere. Each point represents a significant surface area variable, with blue markers indicating right hemisphere regions and yellow markers indicating left hemisphere regions**.** The x-axis lists the regions of interest, while the y-axis represents the F-values**,** highlighting the relative significance of each region in the analysis

**Fig. 6:**
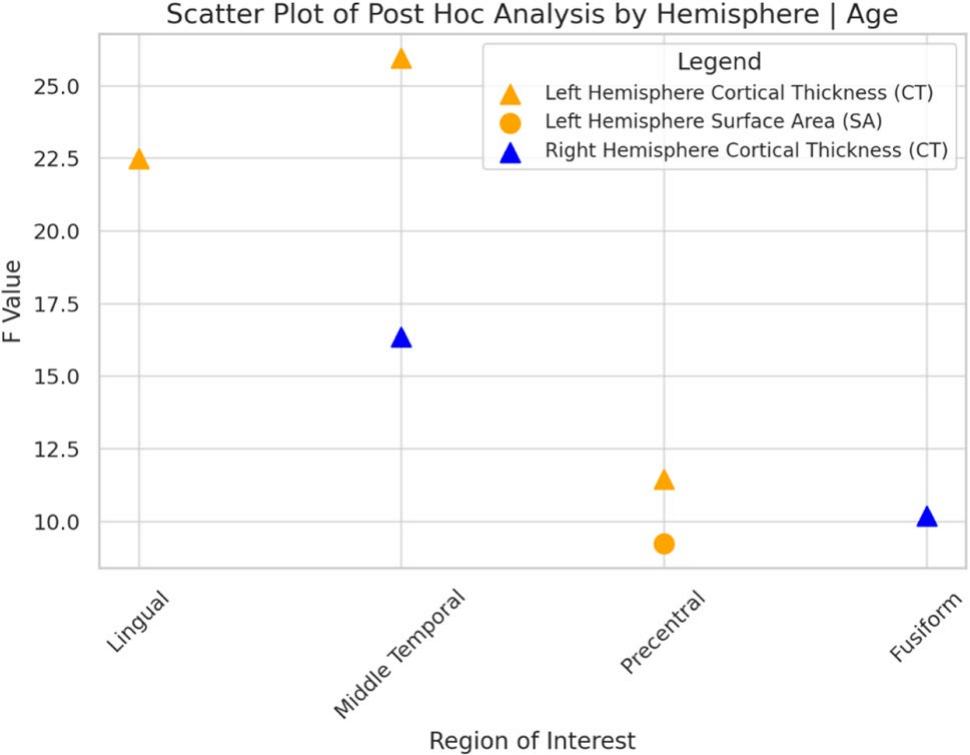
Scatter plot of MANCOVA post hoc analysis for age**,** displaying significant variables stratified by hemisphere and cortical property. The x-axis represents regions of interest**,** while the y-axis displays F-values**,** indicating the statistical significance of each variable. Significant variables are further categorized by hemisphere, with left hemisphere regions represented in yellow and right hemisphere regions in blue**.** Additionally, cortical thickness (CT) variables are marked by triangles**,** whereas surface area (SA) is represented by a single circle

Age significantly explained variance in four CT variables (Fig. 5): LLg (F(1,98) = 22.48), LMdT (F(1,98) = 25.94), RMdT (F(1,98) = 16.34), LPrC (F(1,98) = 11.46), and RFu (F(1,98) = 10.18) and one SA variable, LPrC (F(1,98) = 9.22). ICV significantly explained all SA regions identified in PC1 for both cohorts (), including RParaC (F(1,98) = 59.99), RPO (F(1,98) = 40.67), RPoC (F(1,98) = 40.70), RPC (F(1,98) = 28.77), RPrC (F(1,98) = 52.72), RPc (F(1,98) = 42.99), RRMF (F(1,98) = 87.95), RSF (F(1,98) = 107.23), Rsupm (F(1,98) = 34.76), RTT (F(1,98) = 31.87), RI (F(1,98) = 63.82), LFu (F(1,98) = 42.05), LIP (F(1,98) = 37.79), LIsC (F(1,98) = 49.36), LLOrbF (F(1,98) = 57.15), LMOrbF (F(1,98) = 69.71), LMdT (F(1,98) = 54.59), LPoC (F(1,98) = 32.71), LPC (F(1,98) = 32.11), LPrC (F(1,98) = 43.29), LPc (F(1,98) = 60.58), LRAC (F(1,98) = 52.43), LSF (F(1,98) = 82.10), and LST (F(1,98) = 34.17) Fig. 6.

**Fig. 7:**
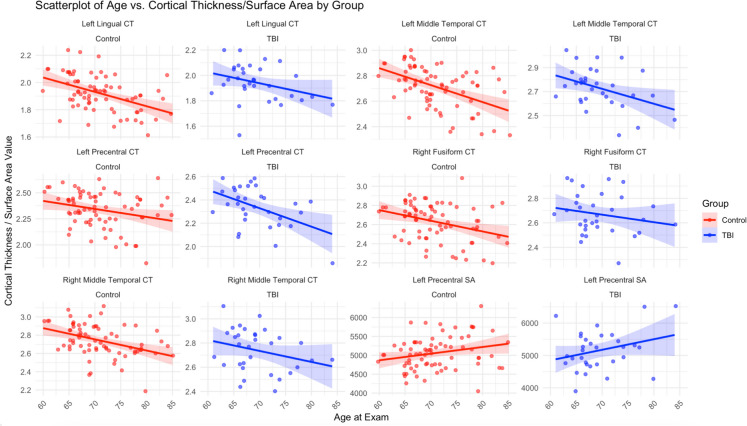
Scatter plot and regression analysis examining variables that remained significant for age after MANCOVA and correction**.** Each panel represents a Cortical Thickness or Surface Area variable**,** plotted against age at exam for both Control (red) and TBI (blue) groups. Linear regression Lines with 95% confidence intervals illustrate the relationship between aging and cortical morphometry across groups

Subsequent exploratory regression analyses further clarified these differences by quantifying the relationship between cortical structures and age within each group (Fig. 7). In the TBI cohort, significant negative associations were observed between age and cortical thickness in the LPrC (β = −0.01582, p = 0.0051, R^2^ = 0.233) and LMdT (β = −0.01246, p = 0.024, R^2^ = 0.158), indicating greater age-related cortical thinning in these regions compared to controls. In contrast, within the Control cohort, significant negative age-related associations were identified in the LLg (β = −0.01054, p < 0.001, R^2^ = 0.212), LMdT (β = −0.01334, p < 0.001, R^2^ = 0.228), and RFu (β = −0.01116, p = 0.004, R^2^ = 0.111), suggesting a broader distribution of cortical thinning with age in controls compared to the more regionally specific effects in the TBI cohort. SA results also demonstrated age-related differences between groups. While the LPrC SA showed a weaker but positive association with age in the Control group (β = 17.63, p = 0.045, R^2^ = 0.055), the corresponding relationship in TBI was not statistically significant (β = 32.66, p = 0.121, R^2^ = 0.078).

## Discussion

### Subthreshold post traumatic stress disorder

Based on the inclusion and exclusion criteria, we expected to see no significant differences between the Control and TBI cohorts in age, education, GDS, and ICV. Despite the exclusion of PTSD diagnosis, a significant difference remained in the CAPS scores between these two cohorts (p = 0.001), suggesting that trauma-related symptoms may still vary between them. Subthreshold PTSD, which is more prevalent than full PTSD in trauma-exposed populations [50], represents a category of individuals experiencing clinically significant symptoms without meeting full diagnostic criteria [51]. Prior research indicates that subthreshold PTSD is associated with worse psychological and behavioral parameters than no-PTSD groups [52], and may influence structural brain changes [53]. Wrocklage et al. (2017) found that greater combat exposure (CES) was associated with significant cortical thinning in non-PTSD participants compared to no such relationships in PTSD participants [53]. Additionally, a multivariate analysis (F(7,54) = 2.39, p = 0.03) confirmed that lower PTSD symptoms correlated with stronger CES-related cortical thinning, suggesting that PTSD may mask the structural effects of combat exposure [53]. Given these findings, the observed demographic differences in our study may reflect variations in trauma exposure and subthreshold PTSD symptoms, which could have implications for cortical morphometry [53].

### Principal component analysis and regression analysis

The PCA analysis reduced the inter-regional covariance across all participants within each cohort, consolidating SA and CT variables to 28 and 39, respectively, to explain the largest portion of variance across original variables [43]. A hemispheric difference in SA distribution emerged, with the TBI cohort showing a trend toward higher left hemisphere (LH) representation of cortical regions and the Control cohort exhibiting more right hemisphere (RH) representation (LH: Control—65. 2%, TBI – 60%; RH: Control – 34. 8%, TBI – 40%). Existing literature on structural and functional asymmetry following brain injury describe a lateralization or asymmetrical phenomenon in which increased connectivity occurs within the RH to compensate for cortical changes within the LH [54, 55]. Consistent with prior studies, the control cohort exhibited more widespread PC1 factor loadings across frontal, parietal, temporal, and limbic regions, while the TBI cohort showed decreased representation across these regions. Specifically, the two cohorts were differentiated by key regions within the DMN. The DMN comprises Pre-Frontal Cortex (PFC), Insula, Inferior Parietal Lobule, Lateral Temporal Cortex, Posterior Cingulate Cortex, Extended Hippocampal Formation, Ventral Medial PFC, and Dorsal Lateral PFC [56]. Regions within the DMN exhibited differential representation across cohorts, with LIP, RPC, and LPC showing high factor loadings in controls but being absent in the TBI cohort, while RI emerged as a significantly different variable between the two cohorts (t = 2. 2619, df = 103, p- value = 0. 02581) and was a high- loading factor exclusively in the TBI group. This pattern of DMN reorganization may reflect cortical SA adaptation to TBI, wherein target regions within the DMN undergo morphometric shifts due to their collective vulnerability to metabolic load in the presence of pathology [43]. Some have hypothesized that neurodegenerative processes may target the DMN structures because of the high metabolic load of maintaining the ‘default state’ of brain function [43].

The CT PCA accounted for greater overall variance in the first dimension for both cohorts compared to SA (SA: Control—44.8%, TBI—40%; CT: Control—46.1%, TBI—48.9%), suggesting that CT may better capture morphometric differences associated with TBI. Similar to SA, a right-biased hemispheric trend emerged in PC1 factor loadings for both cohorts (LH: Control—54.6%, TBI—41.9%; RH: Control—45.5%, TBI—58.1%).

While the control group showed LH dominance in factor loading distribution, the TBI cohort exhibited stronger explained variance from regions within the RH. These findings align with previous research indicating that veterans with a history of TBI are more susceptible to asymmetric cortical thickness changes, particularly in the RH [57].

### Hypothesis one

Our findings support the first hypothesis that TBI survivors exhibit distinct patterns of CT and SA variability compared to age-matched healthy controls, as evidenced by the distribution of factor loadings between cohorts. Regression analyses further highlighted the differences in morphometric organization, with lower residual standard error and higher R^2^ values in the TBI-dominant model, suggesting a more structured pattern of morphometric changes post-injury. Notably, SA changes in TBI followed a more uniform pattern of increased regional values compared to controls, whereas CT exhibited greater variability, with some regions showing thinning and others thickening. The differentiation of factor loadings (Fig. 7) suggests that TBI disrupts normative structural relationships, potentially reflecting adaptive cortical reorganization rather than the universal thinning seen in healthy aging [58] and Alzheimer’s Disease [59]. This systematic alteration in morphometric features post-TBI may provide a basis for improved predictive modeling to enhance classification models that differentiate between pathological and normative aging patterns related to TBI, as has been done in other conditions like Alzheimer’s Disease [60, 61] and Huntington’s Disease [62]. The structured nature of SA changes in TBI survivors, alongside the more heterogeneous alterations in CT, underscores the need for multimodal approaches in predictive modeling that account for both systematic and individualized injury-related cortical adaptations.

### Intracranial volume

ICV is a standard covariate when analyzing CT and SA [29]. In neurodegeneration research, normalization of regional brain volumes by ICV is commonly performed to better estimate the extent of atrophy caused by pathology, independent of intrinsic gender differences or other factors [29]. Likewise, ICV can serve as a proxy for maximum pre-morbid brain volume [63], accounting for head size, a well-known source of between-subject variability [64]. By including ICV as a covariate, our results highlight its role in distinguishing the pathological effects of TBI-related neurodegeneration from natural variations.

After conducting a post-hoc analysis with multiplicity correction, all 28 SA variables that originally loaded onto the first dimension of PC1 in our initial model remained statistically significant (p < 0.001). As ICV is strongly correlated with SA and has been shown to explain a significant proportion of interindividual variance in cortical morphology [20], this outcome was expected. Despite ICV not differing significantly between the two cohorts (p = 0.95), brain regional variance was sufficient to distinguish healthy aging from aging with a history of TBI. In a study of spatial patterns of progressive brain volume loss after moderate-severe TBI among a younger cohort (Mean Age: TBI = 41.55 years, Healthy Controls = 34.22 years), Cole et al. (2018) concluded that atrophy was greatest in white matter compared to grey matter, where the majority (84%) of regions were affected [65]. Our findings contrast with these results, as SA increased across all regions of interest when compared to healthy aging adults. Future studies may investigate the significance of these changes, particularly the mechanisms driving SA increases across brain regions in pathological aging, in contrast to atrophy observed in normal aging. Understanding these compensatory processes may provide insight into how the brain adapts to pathology and could inform targeted interventions for aging individuals with a history of TBI.

To understand why surface area may increase instead of decrease in the presence of TBI pathology during aging, further investigation into the highly heritable nature of surface area is necessary. Specific human mutations have been linked to excessive gyrification of the cortex and increased cortical surface area in the presence of other pathologies, such as Alzheimer’s Disease [66]. Such findings support the need for future investigations into the genetic relationships between total surface area and intracranial volume [20]. Examining the relationship between APOE4, a genetic biomarker associated with increased risk for Alzheimer’s Disease-like neurodegenerative processes, may further explain these associations, highlighting genetic influences distinct from heritable traits.

### Age

Despite no statistically significant cohort differences in age (p = 0.15), age remains a significant factor influencing both SA and CT (p = 0.004). Regions identified in post-hoc analyses highlight those uniquely affected by TBI pathology in relation to age; future research should investigate whether chronological or biological age best explains these differences [67]. Aligned with studies investigating region-specific morphometric changes in chronic TBI, our results indicate that SA and CT respond differently to aging and injury. Specifically, cortical thickness demonstrates an age-related decline consistent with neurodegenerative processes, whereas surface area appears to increase in regions affected by TBI, suggesting compensatory mechanisms or genetic predispositions influencing gyrification and cortical expansion post-injury [68, 69].

### Hypothesis two

Our second hypothesis that age would significantly explain the variability in SA and CT among TBI survivors compared to age-matched healthy controls, was supported by our findings (p = 0.001). Although our analysis did not differentiate quantitatively between biological and chronological age, age remained a critical explanatory factor influencing both SA and CT across groups. Despite a statistically insignificant difference in chronological age between cohorts (p = 0.15), the post-hoc analyses highlighted regions uniquely influenced by TBI pathology in relation to aging. The contrasting responses of SA and CT to age and injury in our study—declines in CT alongside increases in SA—suggest potential compensatory mechanisms or genetic predispositions toward increased gyrification post-injury. These results emphasize the importance of investigating biological age markers and genetic factors in future research to further clarify these relationships.

### Strengths and limitations

A strength of this study is its novel scientific contribution to understanding structural brain changes that can inform and refine intervention strategies, such as the targeted placement of non-invasive brain stimulation and specific cognitive stimulation activities. However, this research has several limitations. First, the sample is composed exclusively of male Vietnam Veterans, which introduces a homogeneity that limits generalizability. The findings may not be directly applicable to women, individuals from more diverse racial or ethnic backgrounds, those with different injury mechanism, severities, or timelines. This restriction should be considered when interpreting the results, as the observed trajectories may not fully represent other TBI populations. Second, the cross-sectional design restricts the ability to draw causal inferences about aging trajectories and prevents assessment of the rate or directionality of cortical changes over time. A longitudinal design would be necessary to disentangle whether observed patterns reflect accelerated aging, compensatory adaptation, or disease-specific remodeling. Finally, while our findings highlight morphometric differences between aging adults and those with a history of TBI, we did not quantify or statistically compare individual differences between regions across these cohorts. Future studies that quantify the amplitude and rate of these regional changes could provide further insight.

### Future considerations

Future research should consider including physiological processes into their analysis, such as glymphatic circulation, microglial reactivity, or other neurovascular implications, to further highlight potential mechanisms underlying observed structural changes. Investigating correlations between these structural changes and significant cognitive or clinical outcomes would provide valuable clinical context. Thirdly, exploring sex differences is crucial to determine the generalizability of these findings. Finally, given that distinct regions emerged uniquely in each cohort in our factor loadings, further studies should assess whether similar regional distinctions characterize aging patterns associated with various neuropathological conditions like Alzheimer’s Disease.

## Conclusions

This study demonstrates that age significantly influences cortical thickness and surface area differently in individuals aging naturally compared to those aging with a history of TBI. The observed increase in surface area in TBI-affected regions suggests possible compensatory neuroplasticity or genetic influences affecting cortical gyrification post-injury. Future research into biological age markers and genetic predispositions may further elucidate these differential responses, enhancing our understanding of neurodegenerative processes in TBI survivors.

## Data Availability

Data used in this study were obtained from the Alzheimer’s Disease Neuroimaging Initiative (ADNI) database (adni.loni.usc.edu). In accordance with ADNI policy, the data are available to qualified investigators upon application and approval of the ADNI Data Use Agreement; the authors are not permitted to redistribute the dataset. No new human data were collected for this study.
